# Topological differences and confounders of mental rotation in cervical dystonia and blepharospasm

**DOI:** 10.1038/s41598-023-33262-4

**Published:** 2023-04-13

**Authors:** Thorsten M. Odorfer, Marie Yabe, Shawn Hiew, Jens Volkmann, Daniel Zeller

**Affiliations:** Department of Neurology, University of Würzburg, 97080 Würzburg, Germany

**Keywords:** Neuroscience, Neurology

## Abstract

Mental rotation (mR) bases on imagination of actual movements. It remains unclear whether there is a specific pattern of mR impairment in focal dystonia. We aimed to investigate mR in patients with cervical dystonia (CD) and blepharospasm (BS) and to assess potential confounders. 23 CD patients and 23 healthy controls (HC) as well as 21 BS and 19 hemifacial spasm (HS) patients were matched for sex, age, and education level. Handedness, finger dexterity, general reaction time, and cognitive status were assessed. Disease severity was evaluated by clinical scales. During mR, photographs of body parts (head, hand, or foot) and a non-corporal object (car) were displayed at different angles rotated within their plane. Subjects were asked to judge laterality of the presented image by keystroke. Both speed and correctness were evaluated. Compared to HC, CD and HS patients performed worse in mR of hands, whereas BS group showed comparable performance. There was a significant association of prolonged mR reaction time (RT) with reduced MoCA scores and with increased RT in an unspecific reaction speed task. After exclusion of cognitively impaired patients, increased RT in the mR of hands was confined to CD group, but not HS. While the question of whether specific patterns of mR impairment reliably define a dystonic endophenotype remains elusive, our findings point to mR as a useful tool, when used carefully with control measures and tasks, which may be capable of identifying specific deficits that distinguish between subtypes of dystonia.

## Introduction

Dystonia is a hyperkinetic movement disorder syndrome which is still considered a rare disease^1^. The key feature of dystonia is involuntary muscle contraction causing abnormal and partly bizarre postures of different body parts. According to body distribution, focal, segmental, multifocal, and generalized forms of dystonia have been defined^2^. Non-motor symptoms like depression, pain and sleep disorders are quite common and, in addition to the motor symptoms, may severely affect patients’ quality of life^3^.

Despite being first described by Oppenheim as early as 1911, over a hundred years ago, the pathophysiology of dystonia remains poorly understood. Three potential pathophysiological mechanisms are frequently discussed (for a review:^4^): loss of cortical inhibition (e. g.^5,6^), synaptic malplasticity (e. g.^7,8^) and altered sensorimotor integration (e. g.^9,10^).

Mental rotation (mR) tests the capacity to imagine rotational movements of objects^11^ and is proposed to serve as a measure of sensorimotor integration^12–14^. Brain areas responsible for planning and execution of movements have been found to be activated during mental rotation suggesting the engagement of higher order motor processes for this task^12,15^. Mental rotation of pictures of body parts and non-corporal objects has already been assessed in focal dystonias, yet this research revealed partly inconclusive outcomes. First, Fiorio et al. were able to show mR impairment for the rotation of hands in focal hand dystonia^16^. The same group demonstrated reduced mR performance for body parts but not for non-corporal objects in cervical dystonia (CD)^17^. Other authors published negative^18,19^ or partly conflicting results^20^, like an impaired mR of capital letters, but not of schematic illustrations of full bodies.

Therefore, an impairment of mR could plausibly serve as a correlate of disturbed sensorimotor integration in dystonia. However, the inconclusive findings published so far necessitate further effort to question their reliability and specificity for a particular dystonic condition. As mR evidently interferes with other conditions but dystonia, aspects like sex^21^, age^22^, and cognitive fitness^23^ also need to be considered when interpreting collected data sets.

To test the hypothesis of mR defining a robust endophenotype of focal dystonia we assessed the task in a cohort of patients suffering from the two most frequent forms of focal dystonia: CD and blepharospasm (BS). Additionally, we aimed at registering potential modulators and/or confounders like cognitive state, dystonic symptom topology and severity, finger dexterity, and working speed systematically.

## Methods

The protocol conformed to the principles of the declaration of Helsinki and was approved by the Ethics Committee of the Medical Faculty at the University of Würzburg. All participants gave their written informed consent for participation in the study.

### Participants

Sixty-three patients were recruited from our outpatient clinic for movement disorders. Patients had been diagnosed with CD (n = 23), BS (n = 21), or hemifacial spasm (HS, n = 19) by a movement disorder specialist. Demographic data are summarized in Tables 1 and 2.

**Table 1:** Summary of demographic and clinical assessments.

	Group	N	Mean	F	*P*
Age (years)	CD	23	59.65	0.79	0.504
	HC	23	59.22		
	BS	21	63.14		
	HS	19	62.26		
9HPT (s)	CD	23	23.45	2.35	0.079
	HC	23	20.42		
	BS	21	21.32		
	HS	19	22.27		
ORTT (s)	CD	23	0.36	2.52	0.064
	HC	23	0.30		
	BS	21	0.36		
	HS	19	0.34		

	Group	N	Mean of ranks	H	*P*
EDU (years)	CD	23	45.24	11.46	0.010
	HC	23	56.48		
	BS	21	35.00		
	HS	19	35.08		
MoCA (score)	CD	23	36.02	20.53	0.0001
	HC	23	61.65		
	BS	21	43.95		
	HS	19	30.08		

CD: cervical dystonia, HC: healthy control, BS: blepharospasm, HS: hemifacial spasm, n: number, 9HPT: 9-hole-peg-test, ORTT: online reaction time test, EDU: education level, MoCA: Montreal Cognitive Assessment.

**Table 2:** Demographic data of cervical dystonia patients and healthy controls.

CD no.	Sex	Age	EDU	MoCA	HC no.	Sex	Age	EDU	MoCA
1	f	50	8	26	18	f	64	12	27
2	m	60	12	27	21	f	54	11	29
3	m	64	10	25	25	m	65	8	27
6	m	64	10	30	26	m	48	9	30
8	f	60	13	30	30	f	47	13	30
9	f	69	9	28	36	f	59	10	30
19	f	50	10	30	44	f	58	12	29
20	m	56	12	27	47	f	53	10	30
22	f	69	10	28	49	f	59	12	30
27	f	69	12	26	52	f	58	10	29
33	f	56	9	25	59	m	77	8	28
38	f	67	9	27	64	f	58	10	29
40	m	32	9	26	65	m	63	9	30
41	f	56	9	26	67	m	72	10	29
51	f	61	7	21	71	f	63	13	30
60	m	68	13	29	72	m	66	13	30
61	f	58	9	25	73	m	67	13	30
63	m	55	9	26	96	f	50	12	30
66	f	64	10	28	97	m	53	12	30
81	f	51	9	30	98	m	64	8	27
87	f	63	9	22	99	f	58	10	30
92	f	62	9	24	100	f	52	10	30
95	f	68	10	30	101	m	54	10	29
Mean		59.7	9.9	26.8			59.2	10.7	29.3
SD		*8.6*	*1.5*	*2.5*			*7.5*	*1.7*	*1.1*

CD: cervical dystonia, No.: number, EDU: educational level (in school years), HC: healthy controls, MoCA: Montreal Cognitive Assessment, f: female, m: male, SD: standard deviation.

All patients were treated with botulinum neurotoxin A injections on a regular basis, while only few received concomitant oral antidystonic therapy or other CNS-effective drugs (for details, see Supplementary Table 1). The experiment was scheduled at least 10 weeks after the last injection date, when no or minor treatment effects remained, as judged both by the experimenter and the patient. Patients with clinically relevant psychiatric or neurological comorbidities were excluded from the study.

Additionally, a control group of 23 healthy volunteers (HC) was recruited. The HC group was matched to the CD group with regard to age, sex, and education level (Table 1). The same matching applied for BS and HS group.

### Clinical assessment

Severity of CD symptoms was assessed via Toronto Western Spasmodic Torticollis Rating Scale (TWSTRS)^24^ by an experienced rater. BS severity was scored on the Blepharospasm Severity Scale^25^, Blepharospasm Disability Index^26^ and Jankovic Rating Scale^27^.

Handedness was defined by means of the Edinburgh Handedness Inventory^28^. The Montreal Cognitive Assessment (MoCA) was used to screen for cognitive deficits^29^, and a MoCA score below 26 points led to exclusion from the HC group. To be aware of unspecific and potentially confounding factors of mR performance, we implemented screening tools for global reaction time in terms of a red light/green light test (here referred to as Online Reaction Time Test [ORTT], University of Washington, https://faculty.washington.edu/chudler/java/stopl.html) and finger dexterity (Nine-Hole Peg Test [9HPT]^30^, average time of right and left hand).

## Mental rotation task

**Figure 1:**
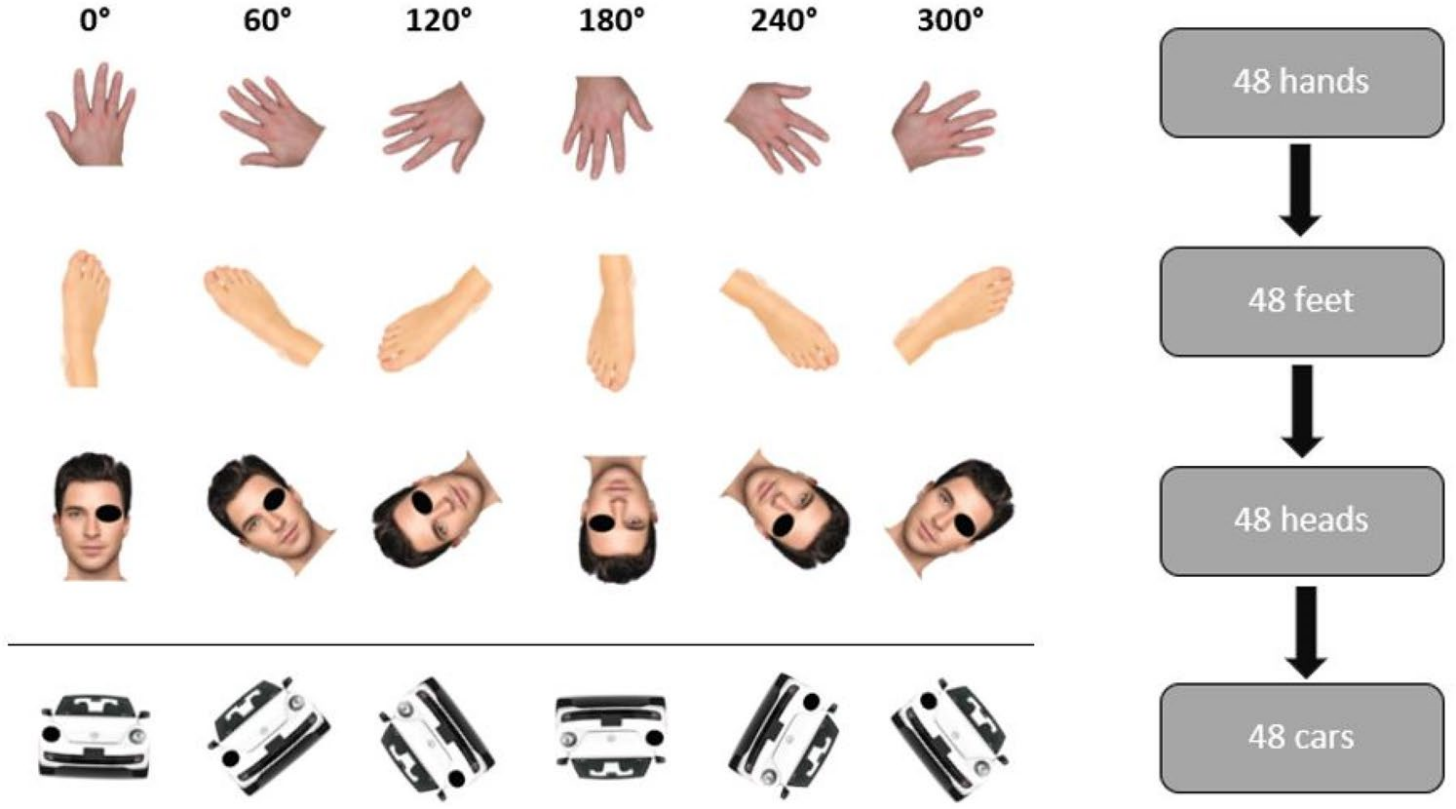
Mental Rotation task: on a screen presented photographs of body parts (in this figure right hands, feet, and heads) and non-corporal objects (here left cars) in different angle.

The mR task was performed in accordance with the procedures described by Fiorio et al.^16,17^. The free software *Open Sesame*^31^ was used to present photographs of body parts (hand, foot and head) and a picture of a car on an ordinary 19 inch computer screen. There were pictures of right and left hands, and feet. As to the images of the head, either the right or left eye was marked with a black spot. The car bore a black mark on either the right or left headlight. The images were presented at six different angles rotated within their plane. Each angle (0°, 60°, 120°, 180°, 240°, and 300°) of each laterality (right and left) was presented four times in a randomized order, but in separate trials for each object (1st run: 48 hands, 2nd run: 48 feet, 3rd run: 48 heads, 4th run: 48 cars; see Fig. 1). Subjects were asked to judge laterality of the presented image by pressing the right and left CTRL keys for right and left responses respectively. Speed and accuracy of responses were recorded. RT for left and right sided stimuli were pooled. Additionally, RT for 60° and 300°, and 120° and 240° were pooled as they are the same angle of rotation in the clockwise and anti-clockwise direction, respectively. To rule out the effect of individual reaction time differences, the difference in RT compared to 0° (no rotation) were used for further analyses.

### Statistical analyses

To evaluate differences in demographic and clinical measures between each group, one-way independent samples analyses of variances (ANOVA), with Tukey post hoc comparisons, were performed with group as the between-subjects variable. Levene’s test was performed to evaluate homogeneity of variances, and when the data violated the assumption of homogeneity of variances, a Welch ANOVA and Games-Howell post hoc test was performed. Data were also tested for normality using the Shapiro–Wilk test, and when the data violated the assumption of normality, the non-parametric one-way ANOVA, i.e. Kruskal–Wallis test was performed.

To evaluate group differences in the mental rotation of the different stimuli, a mixed model ANOVA was performed for each type of stimuli, with group (CD, HC, BS and HS) as the between-subjects variable, and angle of rotation (60°, 120°, and 180°) as the within-subjects variable. We felt justified to conduct analysis of pooled data as paired t-tests revealed no significant differences between clockwise and anticlockwise rotations (60° vs. 300°; 120° vs. 240°) for all stimuli except feet, where rotation performance only at 240° was slightly quicker than 120° (T(84) = 3.77, *p* < 0.001). Concerning equality of laterality of stimuli we ran a mixed model ANOVA as paired t-tests seemed to suggest group differences for hand and car images. After Tukey post-hoc test only one significant finding persisted: mR RT in 180° rotation solely of the right hand stimulus was slower in CD in comparison to HC (F(3, 82) = 2.98, *p* = 0.036). Therefore and for reasons of clarity, we decided to present our data in a pooled form for right and left stimuli as has previously been done^19,20^.

The assumption of normality and sphericity was tested using the Shapiro–Wilk and Mauchly tests, respectively. When the assumption of sphericity was violated, a Greenhouse–Geisser correction was applied. The non-parametric repeated measures ANOVA, the Friedman’s test was used when the data were not normally distributed. Tukey post-hoc comparisons were performed.

One-tailed Spearman’s correlations were performed to assess the relationships between symptom severity as measured by TWSTRS, sum scores of Blepharospasm Severity Scale, Blepharospasm Disability Index, and Jankovic Rating Scale, and response times on the mental rotation task. Spearman’s correlations were also performed to evaluate relationships between ORTT and MoCA with reaction times on the rotation of the different objects.

### Ethical standard

The study was approved by the Local Committee and was conducted in accordance with the ethical standards of the Helsinki Declaration. Informed consent was obtained from all participants after the nature of the study was explained to them.

## Results

### Demographic data

Demographic data of patients and controls are summarized in Table 1, and detailed demographic data of the patients and controls are given in Tables 2 and 3. The groups did not significantly differ in age and sex. However, HC had higher education levels than CD (*p* < 0.010), and HS (*p* < 0.001). They also had higher MoCA scores than CD (*p* < 0.10) and HS (*p* < 0.001).

**Table 3:** Demographic data of blepharospasm and hemifacial spasm patients.

BS no.	Sex	Age	EDU	MoCA	HS no.	Sex	Age	EDU	MoCA
5	f	63	9	27	4	m	62	9	25
12	f	73	8	27	7	m	68	11	22
13	m	47	11	30	10	f	79	8	21
23	f	27	12	30	14	f	70	8	25
24	f	64	9	28	15	f	67	8	27
31	m	49	9	26	16	m	62	9	25
35	m	55	9	26	17	f	77	8	23
39	f	62	10	27	29	m	64	9	27
42	f	57	10	30	34	f	55	9	28
43	f	69	8	28	50	f	60	14	23
46	f	59	10	27	54	f	65	8	22
58	f	74	8	28	56	f	69	10	29
62	f	74	8	27	57	m	67	9	25
77	f	86	8	29	70	m	53	11	29
79	f	73	11	30	74	f	54	9	30
80	f	78	9	25	78	f	66	8	25
82	f	47	10	30	83	f	38	10	30
90	f	68	8	23	84	m	52	9	30
91	f	79	8	23	86	f	55	10	27
93	m	71	8	29					
94	f	51	11	30					
Mean		63.1	9.2	27.6			62.3	9.3	25.9
SD		*13.9*	*1.3*	*2.2*			*9.6*	*1.5*	*2.9*

BS: blepharospasm, No.: number, EDU: educational level (in school years), HS: hemifacial spasm, MoCA: Montreal Cognitive Assessment, f: female, m: male, SD: standard deviation.

### Clinical assessment

Clinical assessments of the patients and controls are summarized in Table 1, and detailed clinical assessment measures are given in Table 4. The groups did not significantly differ on the 9HPT and ORTT.

**Table 4:** Clinical assessment of cervical dystonia and blepharospasm patients.

CD no.	TWSTRS motor	TWSTRS pain	TWSTRS disability	TWSTRS total	BS no.	Severity scale	Disability index	Jankovic scale
1	20	0	11	31	5	6	0	1
2	13	0	3	16	12	9	11	6
3	28	19	15,5	62,5	13	4	0	0
6	21	4	12	37	23	7	4	5
8	20	1	8	29	24	10	7	6
9	21	0	4	25	31	7	2	3
19	4	0	8	12	35	6	3	5
20	13	0	10	23	39	6	1	3
22	24	8	2	34	42	10	22	7
27	17	0	0	17	43	11	8	7
33	18	1	0	19	46	10	16	7
38	20	5	5	30	58	7	10	5
40	9	0	7	16	62	6	1	3
41	19	3	7,5	29,5	77	7	2	4
51	15	0	1,5	16,5	79	7	3	4
60	15	1	4	20	80	6	8	4
61	12	5	12	29	82	4	0	0
63	8	2	8	18	90	13	16	7
66	20	5	10	35	91	6	3	4
81	7	4	9	20	93	6	7	6
87	15	2	9	26	94	7	7	6
92	18	10	15	43				
95	19	0	0	19				

CD: cervical dystonia, No.: number, TWSTRS: Toronto Western Spasmodic Torticollis Rating Scale, BS: blepharospasm.

### Mental rotation task

#### Cervical Dystonia and Hemifacial Spasm patients are impaired on the mental rotation of hands

**Figure 2:**
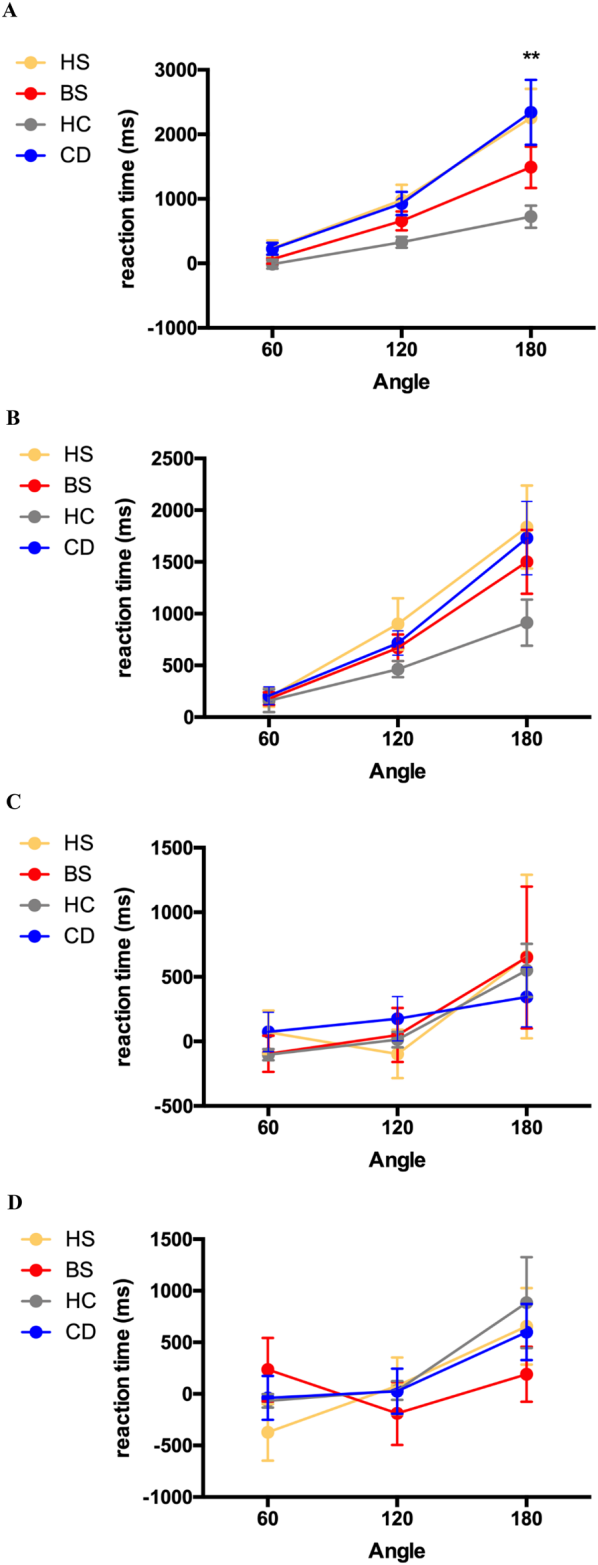
**(A**–**D**) Reaction time relative to no rotation (0°) for each group on the Mental Rotation of (**A**) hands, (**B**) foot, (**C**) head, and (**D**) cars. BS: blepharospasm; CD: cervical dystonia; HC: healthy control; HS: hemifacial spasm, ** *p* < 0.01.

A mixed-model ANOVA revealed significant RT differences between groups for hands (F(3, 82) = 5.04, *p* = 0.003) but not for feet, head and cars (*p* > 0.027; Fig. 2A-D). As differences to the 0° condition are reported here, negative values are possible. Games-Howell post-hoc comparisons confirmed significantly higher RT in CD and HS patients compared to HC only for an angle of rotation of 180° (Fig. 2A).

Furthermore, ANOVA revealed stimulus orientation to be a significant within-subject factor (*p* < 0.001), with increasing RTs with increasing angles of disparity.

There were no significant correlations between RT in the mR task and symptom severity of CD as assessed by the TWSTRS total score (ref. Table 5). The same applied to each of its subscales.

**Table 5:** Correlations between reaction time in the mental rotation task and symptom severity of focal dystonia (Spearman coefficients and corresponding p values).

	Hand	Foot	Head	Car
TWSTRS *total*	− 0.067	0.119	0.193	0.144
	p = 0.760	p = 0.558	p = 0.377	p = 0.512
TWSTRS *motor*	0.230 p = 0.290	0.293 p = 0.175	0.260 p = 0.231	0.220 p = 0.312
TWSTRS *disability*	0.030 p = 0.891	0.201 p = 0.358	0.388 p = 0.068	0.319 p = 0.138
TWSTRS *pain*	− 0.370 p = 0.083	− 0.255 p = 0.241	− 0.123 p = 0.575	− 0.161 p = 0.464
Blepharospasm severity scale	− 0.185	− 0.091	0.201	0.376
	p = 0.422	p = 0.694	p = 0.383	p = 0.093
Blepharospasm disability index	− 0.081	0.040	0.225	0.157
	p = 0.727	p = 0.862	p = 0.328	p = 0.496
Jankovic rating scale	− 0.080	− 0.051	0.182	0.214
	p = 0.730	p = 0.825	p = 0.429	p = 0.352

TWSTRS: Toronto Western Spasmodic Torticollis Rating Scale.

Similarly, there were no significant correlations between RT and clinical measures of BS (sum scores of Blepharospasm Severity Scale, Blepharospasm Disability Index, and Jankovic Rating Scale). Coefficients and corresponding *p*-values are summarized in Table 5.

**Figure 3:**
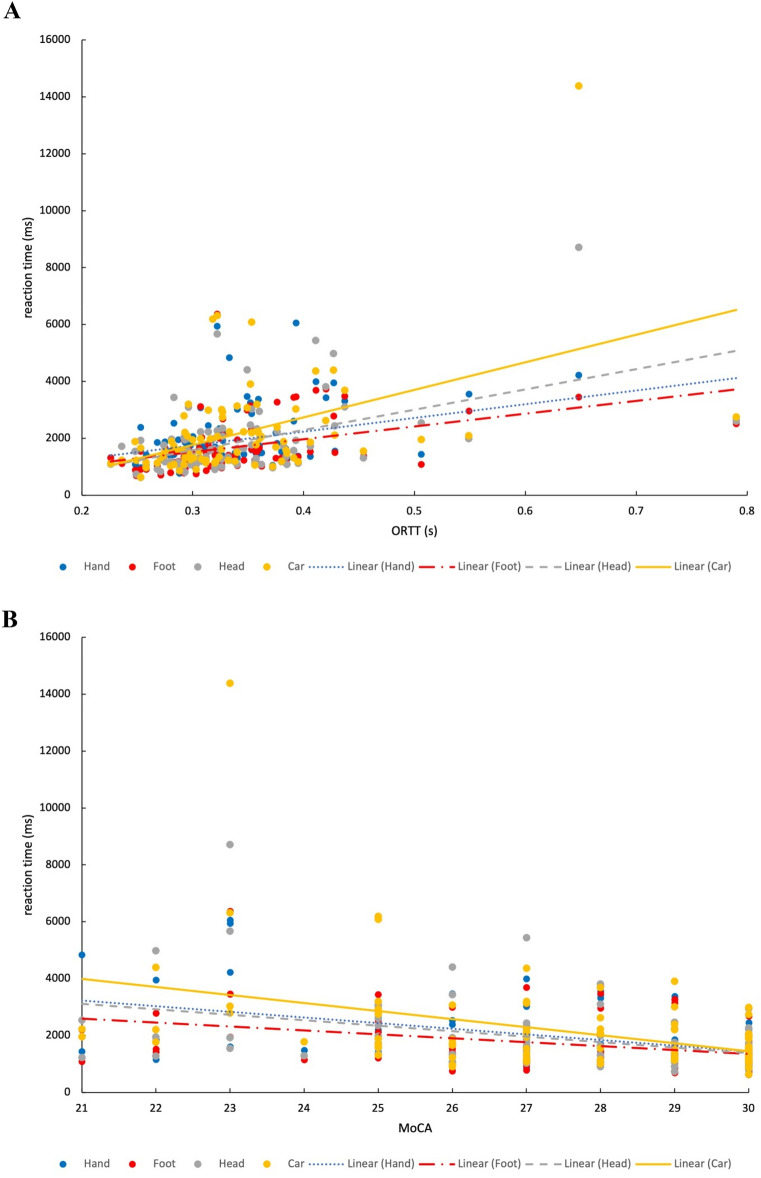
(**A**+**B**) Correlation between mean reaction times of (**A**) Online Reaction Time Test (ORRT) and B MoCA score with Mental Rotation of all stimuli (hand, foot, head, car). Footnote: the correlation remains positive, when removing outliers (mR RT > 7000 ms) from the analysis.

In order to estimate the degree of specificity of RT differences in the mR task, their correlation with global RT (ORTT) was computed. This revealed a moderate positive association for all stimulus objects, which may indicate a contribution of both global RT and mR-specific aspects (hand: r = 0.388,* p* < 0.001; foot: r = 0.473; *p* < 0.001, head: r = 0.423, *p* < 0.001; cars: r = 0.451, *p* < 0.001; see Fig. 3A).

Aside from reaction time, performance in the MR task may be described by the accuracy (ACC) of right/left choices. Accordingly, a mixed-model ANOVA with identical factors (“group”, “stimulus object”, and “stimulus orientation”) was run for ACC as the dependent variable. While there were no group differences in ACC with respect to mR of corporal objects (F(1, 44) = 1.91, *p* = 0.174), HC achieved higher accuracies than CD that were marginally significant (F(1, 44) = 4.134, *p* = 0.048).

#### Cognition as a potential confounder

Comparison of MoCA scores had indicated lower cognitive performance in CD and HS patients compared to HC. Accordingly, significant negative correlations were found between MoCA scores and RT in the mR task across all stimulus objects and all subject groups (hand: r_s_(DF) =  − 0.434, *p* < 0.001; foot: r_s_(DF) =  − 0.419; *p* < 0.001; head: r_s_(DF) =  − 0.363, *p* = 0.001; cars: r_s_(DF) =  − 0.425, *p* < 0.001, see Fig. 3B). Probing single subdivisions of MoCA (*visuospatial, executive, naming, memory, attention, language, abstraction, delayed recall, and orientation*) separately for correlations with mR RT, Spearman’s correlations remain significant except for two conditions (*attention* item with hand stimuli (*p* = 0.145) and *delayed recall* item with head stimuli (*p* = 0.072)).

**Figure 4:**
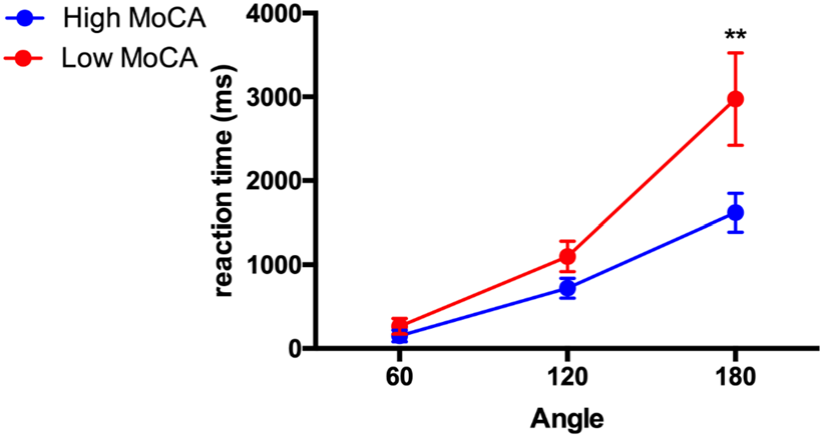
Reaction time on the Mental Rotation of hands relative to no rotation (0°) for patients with (red) and without cognitive impairment (blue).

A comparison of RTs on the mR of hands in patients with and without mild cognitive impairment, defined as a MoCA score lower than 26, revealed that patients with mild cognitive impairment took longer to respond to hand stimuli rotated at an angle of 180° (see Fig. 4).

Thus, in order to exclude cognitive disparities as a confounder in the mR of hands, the mixed-model ANOVA was repeated excluding all patients with MoCA scores < 26 points. In this group of patients and HC without cognitive deficits, there were no significant differences in ORTT (F(3, 63) = 2.63, *p* = 0.111) and 9HPT (F(3, 63) = 2.06, *p* = 0.114), while a group difference concerning lower MoCA scores in CD (new mean/SD 27.9 ± 1.7) persisted after the removal (H = 8.52, *p* = 0.036).

For this subgroup, mixed-model ANOVA showed a significant difference between groups on the mental rotation of hands (F(3, 63) = 4.34*,* p = 0.008). Post hoc comparisons revealed that CD patients needed longer response times than HC and BS *(p* < 0.001).

## Discussion

The present study intended to further probe the concept of mR as an endophenotype of focal dystonia. Hereby, we were able to confirm increased RT in CD and HS compared to HC only on hand stimuli. In contrast, mR RT in patients with BS, another type of focal dystonia, did not differ from healthy controls. Furthermore, assessing potential confounders of mR performance, we detected significant correlations of prolonged mR RT both with reduced MoCA scores and with increased RT in an unspecific reaction speed test. After removing patients with mild cognitive impairment from the analyses, CD continued to show slowed response times to hand stimuli compared to HC while the performance of HS became comparable to HC.

### Mental rotation and cervical dystonia

While performance on mR for HC were well within the range of previously described RTs (between 0.5 and 2.5 s) in healthy subjects, the increased RTs in our CD patients confirm an impairment of mR specifically on hand stimuli in CD. In line with findings by Fiorio et al., we show no differences in performance on mR of non-corporeal objects in our CD patients. Fiorio et al. suggested two main aspects which may contribute to reduced mR speed in dystonia^17^: On the one hand, the dystonic motor syndrome itself might cause delays in mR processing; on the other hand, alterations of the egocentric coordinate system in dystonia are suspected to cause an mR impairment. This interpretation was not only informed by their study in CD patients, but also by a former publication of this group which had shown increased RT in a cohort of patients with writer’s cramp (WC) exclusively during mR of hands while mR of a non-dystonic body region (lower limbs) remained unaffected^32^. The latter was interpreted as an indicator of altered sensorimotor integration due to an aberrant cognitive representation of hand movements in focal hand dystonia^32^. The now replicated observation that mR of body parts is particularly impaired in CD—under the precondition of matched MoCA scores—might be interpreted as to confirm this view. Evidence derived from mR studies in other diseases additionally underpins the assumption that mR comprises basic aspects of sensorimotor integration^33–36^. For instance, in complex regional pain syndrome, mR of hands was prolonged and accompanied by a decrease of fMRI activity in brain networks suspected to be essential for sensorimotor integration^33^.

In contrast, a study by Conson et al.^20^ who performed an mR task of capital letters (and their mirror-reversed forms) and schematic human figures (showing the whole body with hands marked in black to define laterality) in a group of 21 CD patients demonstrated a significant group difference concerning accuracy for letters only, but not for bodies, probably suggesting that CD predominantly affects spatial, but not egocentric object transformation^20^. In support of this, evidence for an impairment of spatial processing in CD is emerging^37–39^. For example, Filip et al. were able to demonstrate elevated fMRI activation in the cerebellum, the parietal cortex and other regions during a visuospatial task as well as reduced connectivity of the cerebellum with basal ganglia structures^39^. This network of cerebellar, thalamic and basal ganglia structures is meanwhile commonly accepted to play a crucial role in the pathophysiology of dystonia^40–44^.

In the present study, we were unable to find any differences in RTs between our CD and HC groups on the mR of foot and head stimuli, nor on non-corporeal objects. One explanation for this is that the differences observed in the RTs of foot and head stimuli, as well as non-corporeal objects were largely driven by differences in reaction times between CD and HC. Indeed, our preliminary analyses revealed longer RTs in CD compared to HC for all stimuli, and at nearly all angles of rotation, as well as in the no rotation (0°) condition. By using only the RT differences between rotation and no rotation in our analyses, we were able to account for the effect of individual reaction time differences. Therefore, in an experimental setting, RT differences are also driven by inter-individual differences in reaction times and these differences must be accounted for in order to identify true differences in mR capabilities.

A second possible confound are the cognitive abilities of the patients in our sample compared to the sample in the study by Fiorio and colleagues. While we evaluated their MoCA scores and reanalysed our data in the subset of patients without cognitive impairment, such measures were not reported by Fiorio and colleagues, while Conson and colleagues included patients with MoCA scores as low as 16. Hence, their sample of CD patients may have included a larger proportion of patients with cognitive impairment driving their poorer performance on mR. Nevertheless, we demonstrated that the impairment in CD on the mR of hand stimuli goes beyond mild cognitive impairment. This suggests that an altered body representation is likely to underlie their impairment on this task. Several different processes contribute to the ability to mentally rotate, namely covert motor rotation, higher order visuospatial thinking and visual object recognition. Removing cognition from possible factors influencing their performance, it is possible that an altered body representation, confined to upper limbs, may hinder their ability to mentally rotate these particular objects. WC patients have also been found to be impaired on the mental rotation of hand^30^. While covert motor processes have been found to share neural correlates with basic movement, processes distinct from basic movements must also underlie these covert operations since CD patients show no differences in dexterity compared to HC, as would be observed in focal hand dystonia, but remain impaired on these covert motor operations.

### A cognitive role in mental rotation

While assessing cognition systematically as a potential confounder of mR in a dystonia cohort, we found a strong relationship between cognitive capability and mR performance. Longer RTs in the mR task were associated with lower MoCA scores. Indeed, mental rotation is a complex task relying on multiple processes including but not exclusive to cognitive processes. Therefore, cognitive abilities must be addressed when evaluating mR capacity. It might be suspected that some cognitive subdomains might contribute more to mR than others, in particular visuospatial, executive, and attentional function could be assumed to be crucial. However, we found significant correlations of nearly all MoCA test subdomains with mR performance. While this precludes conclusions concerning a specific interrelationship between particular cognitive domains and mR performance, it comes as little surprise given the simple design of the MoCA test as a rapid screening tool. Of note, education levels were also correlated with mR performance, but even stronger with MoCA scores (in line with^45^), indicating a kind of circular relationship of these measures. Additionally, cognitive impairment itself may be viewed as a characteristic non-motor feature of dystonia^46^.

Reduced mR capacity has already been identified in several forms of dementia including Alzheimer’s Disease^47^, dementia with Lewy bodies^48^, and ischemic vascular dementias^49^. Some studies even suggest mR as a screening tool for mild cognitive impairment^23,50^. Interestingly, also healthy ageing is associated with altered mR performance^51^; an age-dependent transformation of strategies concerning the rotation procedure is discussed to be the cause^22,52^. Additionally, older persons seem to focus more on accuracy at the expense of longer RT^53^. The latter observation might point to the process of decision-making itself and may also affect mR duration. In CD, this particular phenomenon has already been postulated as the rationale for a reduced capacity in temporal discrimination tasks^54^. Similarly, uncertainty in the context of decision-making during mR could make an important contribution to prolonged RT. As non-motor symptoms including anxiety and depression are common comorbid conditions in CD^46^, and as these mental disorders severely affect decision-making^55^, this additional potential confounder of mR should be kept in mind when planning upcoming trials.

In HS patients without cognitive impairment, and after controlling for simple interindividual reaction time differences, which should account for differences in time required for decision-making, poorer mR performance was no longer observed. This suggests that the impairment of HS on mR is driven purely by cognitive abilities, and not by other symptoms of the disorder. In CD patients without cognitive impairment on the other hand, the impairment for mR of hands remained. This suggests that a deficit other than cognitive impairment and slowed decision making underlies this impairment. Carefully controlling for confounders such as cognitive abilities and interindividual response time differences will allow us to correctly identify which deficit among the multiple processes crucial for mental rotation drives the impairment in our specific population. Only then can we better understand the disorder.

### Differential networks of BS and CD?

To further evaluate the hypothesis of body part-related mR impairment as a general trait marker of focal dystonia, the identical mR paradigm was applied to patients with BS. In contrast to the distinctive finding in CD, BS patients performed non-inferior to a control cohort of HS patients.

It is important to note that we purposely decided to select a clinical rather than a healthy control group. While BS, as a form of focal dystonia, is commonly labeled as central network disorder^56^, HS arises from ectopic or ephaptic excitation due to compression or demyelination of the facial nerve^57^ and is therefore a peripheral neurologic disorder. Apart from these differential pathophysiological roots, the clinical phenotypes of both disorders show a remarkable overlap in terms of frequent involuntary contractions of the orbicularis oculi muscle. Consecutive eye blinks are most likely to interfere with neuropsychological tasks requiring fast recognition of pictures and execution of a simple motor feedback (keystroke). Here, we assume that the extent of this interference does not necessarily depend on whether repeated eye blinks occur uni- (HS) or bilaterally (BS). Indirect support of this assumption might be derived from our finding that symptom severity of BS is not correlated with RT in mR.

By contrast with our observation of an unimpaired mR performance in BS, there is virtually no literature on diverging behavior in CD and BS with respect to other behavioral measures of sensorimotor integration. For example, latencies in a spatial discrimination task were similarly increased in BS and CD^58^. In the same vein, temporal discrimination tasks for visual/tactile^59^ and somatosensory^60^ stimuli revealed significant impairment in CD and BS patients compared to HC, but no differences between the two focal dystonia groups. Beyond that, visuomotor and visuospatial ability is supposed to be disturbed in BS as well as in CD^61,62^. Therefore, despite the clearly diverging phenotypes of focal dystonia, neuropsychological performance seems to be similar in CD and BS, suggesting those findings as a common endophenotype. Moreover, imaging studies suggest that BS and CD pathophysiology may share the same brain network, which essentially contains cortical, basal ganglia, thalamic, and cerebellar structures^63–65^, and so far, there is no convincing evidence of fundamentally differential networks involved in BS and CD pathophysiology.

### A dystonic endophenotype?

Taking these aspects and previous findings into account, there is no simple answer to the question of whether increased RTs in mR are a specific endophenotype of focal dystonia and, further, whether there is a particular relationship between clinically affected body regions and topological patterns of mR impairment, including the divergence between corporal and non-corporal objects.

We were able to replicate the finding of an mR impairment for hands in CD patients with normal MoCA scores, thereby reflecting the particular endophenotypic pattern proposed by Fioro et al. at least partly^17^. However, in the light of literature with opposing results^18–20^ and potential limitations of our approach and Fiorio’s study mentioned above, the specificity of this pattern remains insecure. Notably, Fiorio et al. were able to demonstrate reduced mR performance in patients with CD for the rotation of all body parts and not solely of hands^17^. In contrast, we only found slowed mR in CD patients in a non-dystonic body part, which comprises a topical incongruence of clinical phenotype and neuropsychological endophenotype. Additionally, BS patients did not show comparable alterations of mR, and symptom severity of dystonia did not correlate with the level of mR impairment in the CD group. Altogether, these aspects clearly limit the interpretation of mR defining a distinct dystonic endophenotype. Apart from that, the contribution of unspecific factors, like cognitive state and global reaction speed, appears substantial. While the strong correlation of global reaction speed with mR performance does not exclude an accentuation of speed reduction for the rotation of body parts, the impairment in various speed dependent tasks^61^ may lead to the speculation that a dystonic endophenotype is rather characterized by global deceleration of neurocognitive processes than by selective impairment of the ability to mentally rotate body parts or objects.

Ultimately, the question of whether mR impairment mainly arises from altered sensorimotor integration or from altered spatial processing remains unanswered, yet might be the missing link to understanding the nature of the putative dystonic endophenotype. What seems clear from our study and from several others is that mental rotation is a complex task requiring several crucial processes, and that different performances of the different subtypes of dystonia on specific types of mR tasks may be driven by different processes. When carefully used with several control measures and tasks, mental rotation possibly serves as a useful tool, which could help us understand the neural mechanisms underlying the different dystonia subtypes. Although presenting with similar phenotypes, these subtypes of dystonia may have a different pathophysiology. For example, while WC patients are impaired on rotating hand stimuli both of the affected and unaffected sides, musician’s dystonia patients remain unimpaired on this task^16,18^. Furthermore, WC patients tend to develop a similar dystonia in the unaffected hand when retraining to use the unaffected hand. This suggests that the preexisting abnormalities in WC may be more severe and may bring on dystonia on practice of relatively simple and low-skilled actions. Similarly, the CD patients in our study are impaired on rotating hands. This suggests that while one type of focal dystonia seems largely task specific, musician’s dystonia in this case, there may be more severe underlying abnormalities in the sensorimotor network predating the presentation of the disorder that drives the impairment of CD and WC patients on mR.

Finally, we need to admit that the pathophysiology of the network disorder dystonia still raises plenty of questions to the scientific community. While it appears challenging to get to the heart of the spatial discordance of (clinical) phenotype and (subclinical) endophenotype, we are well aware of the fact that one and the same genotype may be associated with fundamentally different dystonic phenotypes—and vice versa. For example, DYT1 dystonia is well-known to present not only as generalized form but also as multifocal, segmental and even pure focal dystonia^66^. The brain network, which is held to be impaired due to a genetic predisposition, may obviously come along with dystonic symptoms in either one or few body regions or may virtually affect the whole body. This might fuel speculations whether the topology of endophenotype and clinical symptomatology may show similar variability in dystonia.

Further elucidation of the neural underpinnings of mR, for instance by means of functional imaging and TMS inference, is required and under-way^67^. Prior to this, mR performance of patients with (focal) dystonia needs to be interpreted with caution, bearing in mind potential confounding parameters like cognition and speed.

## Conclusion

The question of whether specific patterns of mR impairment reliably define an endophenotype of (focal) dystonia remains partly elusive. However, our findings highlight mR as a powerful tool, when used carefully with control measures and tasks, which may be capable of identifying specific deficits that differentiate different subtypes of dystonia.

## Supplementary Information

Supplementary Information.

## Data Availability

The datasets generated during and/or analysed during the current study are available from the corresponding author on reasonable request.

## Abbreviations

ANOVA: Analyses of variances
BS: Blepharospasm
CD: Cervical dystonia
HC: Healthy controls
HS: Hemifacial spasm
MoCA: Montreal Cognitive Assessment
mR: Mental rotation
ORRT: Online Reaction Time Test
RT: Reaction time(s)
SD: Standard deviation
sec.: Seconds
TWSTRS: Toronto Western Spasmodic Torticollis Rating Scale
WC: Writer’s cramp
9HPT: 9-Hole-Peg-Test
