# Supplementary material for: Topological differences and confounders of mental rotation in cervical dystonia and blepharospasm
Source: Sci Rep. 2023 Apr 13;13:6026. doi: 10.1038/s41598-023-33262-4 (PMC10102235; doi:10.1038/s41598-023-33262-4)
Supplement: Supplementary file 1 — Supplementary Information. [file 41598_2023_33262_MOESM1_ESM.docx]

**Supp. Table 1: Additional clinical data of cervical dystonia and blepharospasm patients**

| **No.** | **YOCO** | **BoNT** | **Antidystonic Drugs** | **Antidepressant Drugs** | **other CNS drugs** | **CD pattern** |
| --- | --- | --- | --- | --- | --- | --- |
| ***1*** | **2016** | **x** |  |  |  | **TC right** |
| ***2*** | **1994** | **x** |  |  |  | **TC left** |
| ***3*** | **1980** | **x** | **clonazepam** |  |  | **TC left** |
| ***5*** | **2000** | **x** |  |  |  | **TC right** |
| ***6*** | **2013** | **x** |  |  |  |  |
| ***8*** | **2009** | **x** |  |  |  | **TC right** |
| ***9*** | **1993** | **x** |  |  |  | **TC right** |
| ***12*** | **2018** | **x** |  |  |  |  |
| ***13*** | **2017** | **x** |  | **sertralin, mirtazpine** |  |  |
| ***19*** | **2011** | **x** |  |  |  | **LC left** |
| ***20*** | **2012** | **x** |  |  |  | **DT** |
| ***22*** | **2011** | **x** |  |  |  | **TC left** |
| ***23*** | **2013** | **x** |  |  |  |  |
| ***24*** | **2006** | **x** |  |  |  |  |
| ***27*** | **2004** | **x** |  |  |  | **TC left** |
| ***31*** | **2014** | **x** |  |  |  |  |
| ***33*** | **2014** | **x** |  |  |  | **DT** |
| ***35*** | **2017** | **x** |  |  |  |  |
| ***38*** | **1990** | **x** |  |  |  | **TC left** |
| ***39*** | **2002** | **x** |  | **venlafaxine, mirtazapine** |  |  |
| ***40*** | **2017** | **x** |  |  |  | **TC left** |
| ***41*** | **1988** | **x** |  |  |  | **TC right** |
| ***42*** | **2017** | **x** | **trihexyphenidyl** |  |  |  |
| ***43*** | **2000** | **x** |  |  |  |  |
| ***46*** | **2017** | **x** | **lorazepam** |  |  |  |
| ***51*** | **1982** | **x** |  |  |  | **TC left** |
| ***58*** | **2001** | **x** |  |  |  |  |
| ***60*** | **1997** | **x** |  |  |  | **TC right** |
| ***61*** | **2009** | **x** | **propranolol** |  |  | **DT** |
| ***62*** | **2005** | **x** | **levodopa** |  |  |  |
| ***63*** | **2000** | **x** |  |  |  | **TC left** |
| ***66*** | **2010** | **x** |  | **escitalopram** | **pregabalin** | **DT** |
| ***77*** | **2011** | **x** |  |  |  |  |
| ***79*** | **2007** | **x** |  | **citalopram** |  |  |
| ***80*** | **2007** | **x** |  |  |  |  |
| ***81*** | **2015** | **x** |  |  |  | **LC right** |
| ***82*** | **2009** | **x** |  |  |  |  |
| ***87*** | **1995** | **x** |  |  |  | **DT** |
| ***90*** | **2017** | **x** |  | **mirtazapine** |  |  |
| ***91*** | **2015** | **x** |  | **mirtazapine** |  |  |
| ***92*** | **2018** | **x** |  |  |  | **LC left** |
| ***93*** | **2019** | **x** |  |  |  |  |
| ***94*** | **2019** | **x** |  |  | **tramadol** |  |
| ***95*** | **2015** | **x** |  |  |  | **TC left** |

**Abbreviations:** CD: cervical dystonia, No.: number, YOCO: year of clinical onset, BoNT: botulinum neurotoxin, CNS: central nervous system, TC: torticollis, LC: laterocollis, DT: dystonic tremor.
